# Ranula as the First Symptom of HIV Infection in Young Patients

**DOI:** 10.1155/2021/8874662

**Published:** 2021-06-29

**Authors:** X. Vanden Eynden, C. Bouland, D. Dequanter, M. Gerbaux, S. Kampouridis, E. Boutremans, I. Loeb

**Affiliations:** ^1^Department of Stomatology and Maxillofacial Surgery, Université Libre de Bruxelles (ULB), CHU-Saint-Pierre, Rue Haute 322, Brussels 1000, Belgium; ^2^Department of Pediatrics, Queen Fabiola Children's University Hospital, Université Libre de Bruxelles (ULB), Avenue Jean-Joseph Crocq 15, Brussels 1020, Belgium; ^3^Department of Radiology, Université Libre de Bruxelles (ULB), CHU-Saint-Pierre, Rue Haute 322, Brussels 1000, Belgium

## Abstract

**Introduction:**

Oral manifestations are often the earliest HIV signs. Salivary gland diseases are a common form of HIV expression. A ranula can occur in association with HIV. However, this manifestation is rarely considered as the disease sentinel sign. We present two cases of children consulting for a ranula, leading to the diagnosis of a previously unknown HIV infection. *Case Reports*. Two children, respectively, 5 and 13, were treated for a ranula by marsupialization. Relapse occurred in both cases, and thereafter, a ranula excision was performed. While the follow-up was uneventful, HIV infection was diagnosed during the patients' care. The only sign or symptom observed was the ranula. A routine HIV testing of ranula patients would have allowed earlier care.

**Conclusion:**

Routine HIV testing of patients with a ranula is justified and may be recommended, especially for children. Ranula excision associated with the sublingual gland resection is suggested in order to avoid recurrence.

## 1. Introduction

Oral manifestations are often the earliest signs of HIV infection [1]. Salivary gland diseases or HIV-associated salivary gland disease is a common sign of the pathology [1, 2]. These diseases include neoplastic and nonneoplastic diseases. The neoplastic disease group includes Kaposi sarcomas and salivary gland lymphomas. The nonneoplastic disease group contains benign lymphoepithelial lesions (BLELs), cystic lymphoid hyperplasia (CLH) or benign lymphoepithelial cysts (BLECs), diffuse infiltrative lymphocytosis syndrome, and parotid lymphadenopathy [2]. Ranulas or retention cysts are also described in association with HIV infection. However, the association between ranulae and HIV status is not well understood, especially when these lesions are the initial symptoms of HIV infection in young patients. We present two cases of young patients consulting for a ranula with an unknown HIV infection.

## 2. Case Report 1

A five-year-old female Cameroonian patient, born in January 2007, was referred to the Stomatology and Maxillofacial Surgery Department, in August 2012, for a painless swelling of the floor of the mouth. This swelling had been evolving for one week, accompanied by deglutition problems, without fever or any other clinical manifestation. Except for a Bactrim allergy, the patient did not present any known medical history, especially no growth retardation and no history of recurrent or severe infections nor any hospitalization or blood transfusion. No HIV checkup had ever been performed on the child, even though both parents were HIV positive.

**Figure 1:**
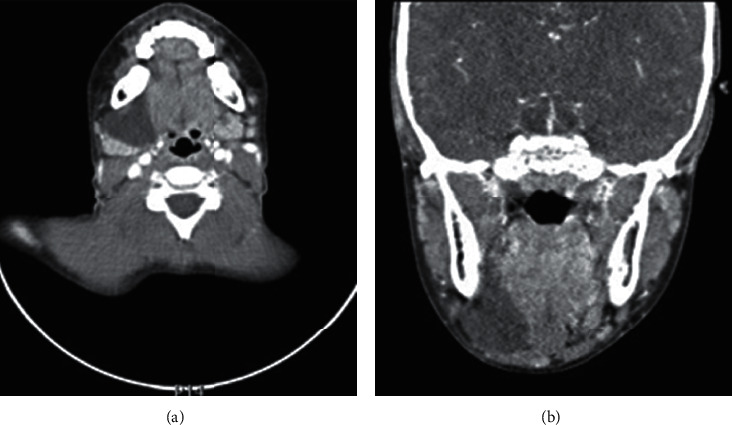
(a, b) Injected cervicofacial CT-scan demonstrating the presence of a well-delineated liquid lesion of 25 × 35 × 29 mm.

The clinical examination showed a painless blue swelling of the floor of the mouth that had appeared one week before. The biological analysis only identified abnormal coagulation results. The clinical diagnosis concluded to a ranula. A marsupialization was proposed. However, the treatment was postponed until complete hemostasis work-up. The patient travelled back to Cameroon and came back a year later with the same lesion. In July 2013, coagulation was within normal range with no other biological abnormality observed, and the marsupialization of the ranula was performed. Postoperative follow-up was uneventful. One month later, the patient presented a pulmonary infection requiring hospitalization and underwent a complete checkup, including routine blood tests as well as microbiological test (tuberculin intradermo reaction, serology for *Mycoplasma pneumoniae* and *Chlamydia pneumoniae*, and nasopharyngeal aspiration with testing for common epidemic viruses). After a thorough anamnesis, the patient's mother recounted that her daughter presented a chronic cough for a long time. During the medical investigations, HIV serology was performed, demonstrating an HIV infection. The patient had 172 CD4 cells/mm and a viral load of 64000 copies/mL at the time of diagnosis. An opportunistic infection was suspected, and however, no germs were highlighted. The patient was immediately treated with highly active antiretroviral therapy (HAART). In August 2014, the patient came back to the department with a right submandibular swelling that had been evolving for six months. The clinical examination revealed a massive and slightly painful swelling in the right submandibular region. A radiography, an ultrasound, and a CT-scan (Figures 1(a) and 1(b)) were undertaken, confirming the presence of a liquid cyst of 25 × 35 × 29 mm, located in the right submandibular region and pushing back the homolateral submandibular gland. Subsequently, the excision of the cystic lesion associated with the resection of the sublingual and submandibular glands was performed. The postoperative course was favorable. Since then, no ranula recurrence has been observed and the patient comes back every year to Belgium for her HIV follow-up. The patient is now 14 years old and is being treated with anti-HIV tritherapy. The last blood sample showed an undetectable viral load. No opportunistic infection has occurred. The anatomopathological examination of the two fragments, of, respectively, 3 × 2 × 1.3 cm and 3.8 × 2.5 × 2.5 cm, revealed cellular tissue containing a few lymphoid isles, with rare images of lymphoepithelial complexes and the presence of an extensive cavity full of mucin. This cavity was delimited with a fibrous deepithelized wall. The anatomopathological results confirmed the diagnosis of a ranula.

## 3. Case Report 2

**Figure 2:**
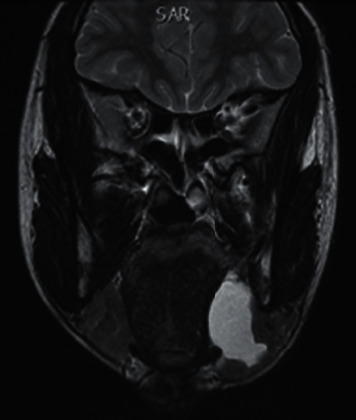
Magnetic resonance imaging T2 sequence, the cystic process of 31 × 31 × 17 mm occupying the lingual pelvis.

A thirteen-year-old female Congolese patient, born in August 2000 and living in Belgium since the age of four, was referred to the Stomatology and Maxillofacial Surgery Department, in January 2014, for swelling of the floor of the mouth associated with a submandibular mass. Both appeared a few days previous, along with dysphagia. The patient had no other symptoms. The patient was in good health, height and weight were in the normal percentile range, and she presented no medical history, nor allergies. The patient had never been hospitalized, never had a severe or recurrent infection, nor received a blood transfusion. The clinical examination showed a soft painless blue swelling of the left side floor of the mouth. Except for the left submandibular mass, no cervical lymphadenopathy was palpated. Medical imaging, including an ultrasound and magnetic resonance (Figure 2), was performed confirming the presence of a cystic lesion of 31 × 31 × 17 mm. The patient's father was HIV positive, and the mother was HIV negative in 2012. The patient has four brothers, all HIV negative. A preoperative HIV serology was performed, due to the familial context and clinical presentation, and led to the diagnosis of an HIV infection. The patient had 477 CD4 cells/mm and a viral load of 8310 copies/mL at the time of diagnosis. No HIV treatment was prescribed at that time. A marsupialization of the lesion was performed. Unfortunately, relapse of the lesion occurred three months later. The patient benefited from a resection of the cystic lesion and the sublingual gland. The anatomopathological examination of the 4.5 × 1.5 × 1 cm fragment revealed a seromucous salivary gland characterized by canals dilatation, containing mucin and a discreet chronic inflammatory infiltrate without lymphoid formation that could fit in a mucocoele context. The postoperative follow-up was uneventful. No recurrence of the lesion was observed since then. Since 2017, the patient has been treated with anti-HIV tritherapy. The last blood sample showed an undetectable viral load. The patient is currently treated with an integrase inhibitor.

## 4. Discussion

Ranulas are mucocoeles or retention cysts, described among the oral manifestations in association with HIV infection [2]. They are formed by the extravasation of mucus from the sublingual gland, presumably due to continued production of saliva in the presence of ductal obstruction [1]. Usually, mucocoele or ranula occurs after a trauma. However, no history of trauma in the current cases nor in other studies [3] could explain the ranula development. Different hypotheses were raised to explain its physiopathogenesis. A persistent chronic inflammation caused by HIV could lead to the obstruction of a small duct, which followed by distension could lead to a subsequent rupture and mucus extravasation into the surrounding tissues [4]. Syebele and Munzhelele discussed that ranulae could be an HIV-related salivary gland disease (HIV-SGD) based on the similarities with BLEC [4], as diffuse lymphoid infiltrates are observed in salivary gland parenchyma in HIV-infected patients. Previous studies considered that diffuse infiltrative CD8 lymphocytosis syndrome (DILS) could play an etiological role in the occurrence of BLEL or BLEC of parotid salivary gland. Indeed, large amounts of HIV-1 p24 and RNA copies in the fluid content of BLEC of parotid gland in HIV-infected patients have been demonstrated previously. Based on these results, the authors hypothesized that the BLEC was the HIV-1 virus reservoir. However, HIV-1 virus has also been observed in the fluid content of ranulae in HIV-infected patients. Nevertheless, there is no evidence to support potential physiopathogenesis [4].

The ranula's treatment has always been controversial. Different strategies have been proposed: marsupialization with or without open packing, excision with or without resection of the sublingual gland, and laser excision. Lai and Poon [5] suggested that carbon dioxide laser excision is a safe method with minimal or no recurrence of the disease. In the present study, a marsupialization was executed in the two cases; unfortunately, a recurrence of the lesion appeared in both cases. However, the marsupialization stitch-and-stab technique has been successful without the ranula's recurrence [6]. No relapse occurred when the excision of the lesion is associated with the resection of the sublingual gland. Syebele and Bütow. have studied the effect of HAART, the recommended treatment for CLH, on ranulae showing limited success [7]. There is no evidence to support a ranula's treatment using HAART.

Both patients did not know their HIV status at the first pediatric consultation. Their HIV status remained unknown until the ranula manifestation, and initially, the ranula was their only complaint despite the HIV infection. The literature rarely describes these lesions as the initial symptoms or signs of an HIV infection [7–9]. In 2010, Syebele and Bütow [7] suggested that ranulae could be considered as the initial symptoms of an HIV infection, on a sample of 48 patients. Thirty-three had an HIV infection, even though nineteen were considered “healthy.” Based on those results, the authors concluded that routine HIV testing in all patients with oral mucocoeles and ranulae is justified and should be recommended. The ranula patients with an HIV infection, in Syebele and Bütow [7] and Chidzonga and Rusakaniko [9], were predominantly aged between zero and 20, like our two patients, respectively, 5 and 13 years old. The high prevalence of HIV among the young ranula patients could be attributed to the mother-to-child transmission [6]. Since buccal ranulae are rare in patients between zero and 15 years [1] (0.08% overall prevalence), HIV testing should be encouraged, especially for young patients. Routinely, HIV serology is not prescribed before a surgical procedure. Both patients were tested for different reasons. The first patient presented a severe pulmonary infection, and both parents were HIV positive. In the second case, an HIV serology was prescribed before the surgical procedure due to similarities with the first patient: a ranula patient with an HIV positive relative.

In 2015, Syebele and Munzhelele highlighted that HIV positive patients present a significantly higher risk (*p* < 0,001) of developing a ranula [4]. However, they noted that most cases originated from the Sub-Saharan Region. The studies conducted in other parts of the world did not highlight any link between ranulae and an HIV infection [4]. To our knowledge, only one case of ranula has been described, in Europe, in a young patient with an unknown HIV [1]. Nevertheless, the patient had Sub-Saharan roots as in the present study.

Furthermore, Kamulegeya and Okello [10] suggested that ranulae could also be a sign of decreased immunity within the HIV/AIDS disease, 23.8% patients had <200 CD4 cells/mm, and 40.5% patients had >500 CD4 cells/mm, at the time of diagnosis. However, no statistically significant differences were observed between CD4 cell count and ranulae presence [6]. In the present study, both patients presented a ranula, and at the initial diagnosis, the CD4 cell count varied between 172 and 477. Regardless of the immunity level, the presence of a ranula suggests performing HIV testing.

In conclusion, routine HIV testing of patients with a ranula is justified and may be recommended, especially for young patients. Excision of the ranula associated with resection of the sublingual gland is suggested in order to avoid recurrence.

## Data Availability

The data used to support the findings of this study were extracted from the Saint-Pierre Hospital database and are available on request.
